# Liver Innervation in Health and Disease: Neuroimmune–Neurovascular Interface and Future Therapeutic Implications

**DOI:** 10.3390/biomedicines13123091

**Published:** 2025-12-15

**Authors:** Marcello Trucas, Denis Barry, Melissa J. Conroy, Michela Vincis, Andrea Diana, Claudio Intini, Pietro Gobbi, Clara Gerosa, Daniela Fanni, Andrea Perra

**Affiliations:** 1 Unit of Citomorphology, Department of Biomedical Sciences, University of Cagliari, Cittadella Universitaria, 09042 Monserrato, Italy; diana@unica.it (A.D.);; 2 Discipline of Anatomy, School of Medicine, Trinity College Dublin, 152–160 Pearse St, D02 R590 Dublin, Ireland; debarry@tcd.ie (D.B.); meconroy@tcd.ie (M.J.C.); 3 Cancer Immunology Research Group, Trinity St. James’s Cancer Institute, Trinity College Dublin, James Street, D08 NHY1 Dublin, Ireland; 4 Unit of Anatomical Pathology, Department of Medical Sciences and Public Health, University of Cagliari, Cittadella Universitaria, 09042 Monserrato, Italy; michela.vincis86@unica.it (M.V.); clara.gerosa@unica.it (C.G.); daniela.fanni@unica.it (D.F.); 5 Tissue Engineering Research Group, Department of Anatomy and Regenerative Medicine, Royal College of Surgeons in Ireland, 123 St Stephen’s Green, D02 YN77 Dublin, Ireland; 6 Unit of Electron Microscopy, Department of Biomolecular Sciences, Campus Scientifico “Enrico Mattei”, University of Urbino Carlo Bo, Via Ca’ le Suore 2—Località Crocicchia, 61029 Urbino, Italy; pietro.gobbi@uniurb.it; 7 Unit of Oncology and Molecular Pathology, Department of Biomedical Sciences, University of Cagliari, Cittadella Universitaria, 09042 Monserrato, Italy; andrea.perra@unica.it

**Keywords:** hepatic nervous system, neuroimmunity, regenerative medicine, neurovascular regulation, liver-brain axis in cirrhosis, neuromodulatory therapy, organoids, AI

## Abstract

The liver is intricately innervated by sympathetic, parasympathetic, and sensory fibres, forming a dynamic neurovascular and neuroimmune network that regulates hepatic function and contributes to disease pathogenesis. While traditionally underexplored, hepatic innervation is now recognised as a key modulator of metabolic homeostasis, immune surveillance, and vascular tone. Historically, the liver was not considered a major target of neural regulation, but recent advances in neurology and imaging have revealed complex and dynamic interactions between neural circuits and hepatic functions. This review provides a comprehensive overview of liver innervation, detailing its anatomical organisation and functional roles in both physiological and pathological contexts. We investigate the role of liver innervation in shaping immune responses, particularly in the context of metabolic dysfunction-associated steatotic liver disease, alcohol-associated liver disease, and autoimmune liver diseases, including autoimmune hepatitis and primary biliary cholangitis. Special attention is given to the neuroimmune crosstalk that governs inflammation, fibrosis, malignancy, and tissue remodelling. Furthermore, we examine how neural inputs influence hepatic blood flow, sinusoidal endothelial function, and portal hypertension, highlighting the interplay between neural and vascular systems. We highlight neuromodulatory approaches, including vagus nerve stimulation and other agents to modulate liver inflammation, vascular dysfunction, and immune dysregulation. Finally, we discuss emerging research models, including liver organoids, Artificial Intelligence-based digital twins and biomaterials as innovative platforms designed to study neural-liver interactions and test new therapeutic strategies. By integrating neuromorphology, immunology, and hepatology, this review aims to advance our understanding of liver innervation as a central player in hepatic health and disease and to identify novel targets for therapeutic intervention.

## 1. Neuroanatomy of the Liver

The liver receives a rich supply of nerve fibres from both the sympathetic and parasympathetic branches of the autonomic nervous system, as well as sensory fibres [1]. These fibres form a complex network that innervates the hepatic artery, portal vein, bile ducts, and liver parenchyma [1,2,3,4]. Sympathetic innervation originates from the celiac ganglia and travels along the hepatic artery, releasing neurotransmitters such as norepinephrine that influence vascular tone and metabolic processes [1,5]. Parasympathetic innervation is provided by the vagus nerve, which releases acetylcholine and other neurotransmitters to modulate liver functions [6,7].

Sensory innervation, primarily from the vagus nerve and spinal afferents, provides feedback to the central nervous system about the liver’s status, including pain and stretch sensations [8]. Advanced neural mapping techniques, such as viral tracers and optogenetics, have been employed to elucidate the precise anatomical organisation and functional roles of these neural pathways [9,10].

The autonomic nervous system plays a central role in regulating liver physiology, including microcirculation, hepatocyte metabolism, and bile secretion. This regulation is mediated by both sympathetic and parasympathetic fibres, whose anatomical distribution varies across species. While the liver’s microscopic architecture is generally conserved among mammals, the pattern of innervation within the liver lobule is highly species-specific. In most mammals, parasympathetic fibres are confined to the portal triads, whereas sympathetic fibres, when present in the parenchyma, extend toward the sinusoids [11]. Humans exhibit a denser network of intralobular and intrasinusoidal nerve fibres compared to rodents like rats, which show only sparse parenchymal innervation. However, the portal tracts in rats are relatively well-innervated, comparable to those in humans [7,12].

The liver’s innervation has been widely demonstrated by means of histochemistry, immunohistochemistry, and electron microscopy. Starting from the hepatic hilum, a complex network of autonomic fibre branches spreads through the stroma of the organ along the branches of the portal vein, hepatic artery, and bile ducts [13]. Along this pathway, contiguities exist between nerve fibres and MHC class II-expressing dendritic cells (DCs). These relationships could be represented by appositions of identifiable DCs to the basal lamina of unmyelinated nerves, although without membrane specialisations [14]. However, DCs reportedly display intercellular connections with fibroblasts [15,16]. At the periphery of the lobule, the nervous network spreads along the sinusoids until it reaches the centrolobular vein and the smooth muscle structures associated with it [13]. Along the sinusoids, and specifically in the spaces of Disse, both adrenergic [17] cholinergic [18] and peptidergic [19] axon terminals, in the cytoneural junction with hepatocytes, have been described. Intraparenchymal sensory nerve terminals have also been identified within the spaces of Disse and the interhepatocytic space [20]; moreover, a peptidergic innervation of the liver lymphatic capillaries was demonstrated by Ito et al. in 1990 [21].

**Figure 1:**
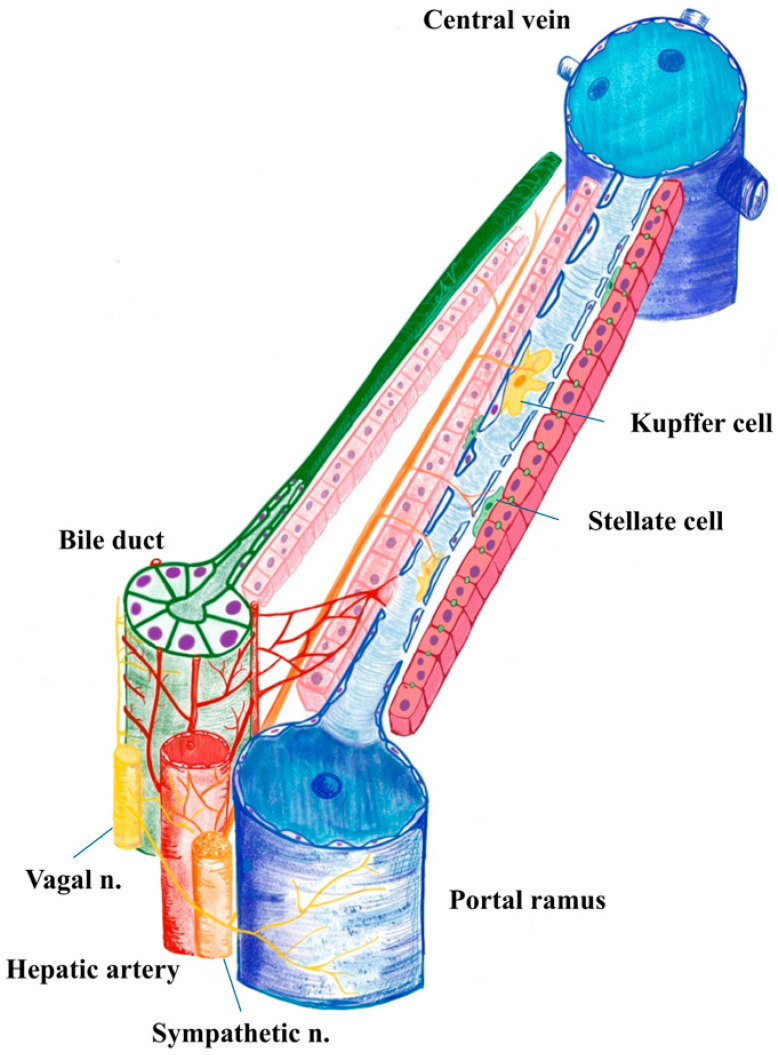
Human liver acinus illustrating the portal triad and central vein. Parasympathetic fibres predominantly course within the portal spaces, accompanying branches of the portal vein, hepatic artery and bile duct with the Peri Biliary Plexus (which, after surrounding the bile duct, pours into the sinusoid). In contrast, sympathetic fibres penetrate the hepatic parenchyma, where they establish neuroimmune interactions with sinusoidal resident macrophages (Kupffer cells), modulating local immune responses and microcirculatory dynamics (used Infinite Painter, v. 7.1.18, by M.V.).

While direct anatomical or synaptic connections between Kupffer cells (KCs) and nerves are not yet clearly established, there is strong evidence for functional neuroimmune interactions (Figure 1). Kupffer cells are responsive to neural signals and may play a role in integrating hepatic immune responses with autonomic nervous system activity [22]. Also, the proximal human biliary tree (interlobular bile ducts, bile ductules and canals of Hering) presents a diffuse juxtaposition between nerves (predominantly adrenergic) and the biliary basal membrane [23].

Taken together, these studies demonstrate that no single, stromal or parenchymal, liver component is deprived of efferent and afferent nerves or nerve influence. The “sensory system” of the liver is based on the relationship between the endothelial cells of lymph and blood capillaries (flows, pressures) [24], while the liver’s “motor system” is associated with direct neural control of hepatocytes in metabolic activities, bile production and conduction, blood pressure, inflammatory control, and stromal fibroblast control. The relatively fast regeneration of liver nerves after experimental denervation [25], indirectly underlines the crucial role of innervation for liver physiology. Furthermore, the loss of hepatic innervation, which is perpetuated by chronic inflammation and fibrosis during cirrhosis [19,26], may represent a worsening element of the degenerative process.

## 2. Neural Regulation of Liver Functions

### 2.1. Parasympathetic Innervation and Regeneration

Parasympathetic innervation, primarily via the vagus nerve, modulates cholangiocyte function, including secretion, growth, and apoptosis, and stimulates liver progenitor cells. Under normal conditions, these progenitor cells receive cholinergic input through acetylcholine binding to M3 receptors, a process regulated by hepatocyte-derived acetylcholinesterase [27]. When hepatocytes undergo necrosis, reduced acetylcholinesterase (AChE) activity can enhance cholinergic stimulation of the progenitor compartment, facilitating parenchymal regeneration [28]. A study involving mouse hepatocyte rat liver cell hybrids demonstrated that hepatocytes could secrete acetylcholinesterase (AChE), the enzyme responsible for breaking down acetylcholine [29]. Additionally, the vagus nerve contributes to immune tolerance in the liver, whereby acetylcholine released from vagal terminals inhibits pro-inflammatory cytokine production via α7 nicotinic acetylcholine receptors (α7nAChR), expressed on Kupffer cells and other immune cells [30]. Indeed, recent research has shown that vagus nerve stimulation also activates the cholinergic anti-inflammatory pathway in the regulation of systemic inflammation, and this is not limited to the hepatic compartment [31,32]. Activation of α7nAChR initiates intracellular signalling cascades that inhibit NF-κB activation, thereby reducing the transcription of pro-inflammatory cytokines. While the anti-inflammatory role of α7nAChR is well established, its involvement in other immune processes, such as the recruitment and trafficking of monocytes and macrophages to sites of inflammation, remains incompletely understood [27,33]. Also, the intensity of cholinergic signalling influences cytokine production and leukocyte trafficking, with direct implications for hepatic inflammation and repair [34,35].

Additionally, the liver’s circadian rhythms are influenced by neural signals, which help synchronise metabolic processes with the body’s overall circadian cycle. The hepatic vagus nerve (HVN) plays a key role in transmitting clock-dependent signals between the liver and brain. Disruption of liver clock genes (e.g., *REV*-*ERBα*/*β* or *BMAL1*) alters feeding behaviour and metabolic rhythms in mice. These changes are mediated through the HVN, which relays liver-derived signals to the brain [36].

Partial hepatectomy in rats leads to an immediate reduction in portal tract innervation in the remaining liver tissue. However, a compensatory hyperinnervation is observed at the hepatic hilum within a week [6,37]. Liver regeneration in this context involves the proliferation of all mature cell types, including hepatocytes and biliary epithelial cells. This raises the question of whether cholinergic fibres adaptively follow the expansion of cholangiocytes during regeneration, and further work is warranted to elucidate the sequence of these biological events [6].

### 2.2. Sympathetic Innervation

Neural sympathetic inputs play a crucial role in regulating various liver functions, including glucose and lipid metabolism, and bile secretion. Sympathetic activation generally promotes glycogenolysis and gluconeogenesis, increasing blood glucose levels, while parasympathetic activation enhances glycogen synthesis and lipid storage [7]. This neural regulation also extends to bile secretion, whereby parasympathetic stimulation promotes bile flow, while sympathetic stimulation inhibits it [1].

Electron microscopy studies have revealed noradrenaline-immunoreactive fibres in the human liver parenchyma, a feature absent in rats. In humans and other species, nerve fibres have been observed in direct contact with hepatocytes, an arrangement not found in rats or golden hamsters. This species-specific difference suggests more complex sympathetic innervation in the human liver, potentially contributing to differences in hepatic neuroregulation and disease susceptibility [38].

Hepatic stellate cells (HSCs) or Ito cells, located in the space of Disse, between hepatocytes and sinusoidal endothelium (Figure 1), are the liver’s principal fibrogenic cells, and are influenced by sympathetic nerve fibres. These cells express adrenergic receptors and catecholamine biosynthetic enzymes, allowing them to respond to norepinephrine (NE) and other neurotransmitters. In the disease setting, NE promotes HSC proliferation and activation, contributing to fibrosis and cirrhosis. Conversely, blocking adrenergic signalling with antagonists or sympathectomy reduces fibrosis severity [39,40].

The sympathetic nervous system, via α1-adrenergic receptors, contributes to liver inflammation by activating Kupffer cells and inhibiting hepatic progenitor cell (HPC) proliferation, leading to increased secretion of IL-6 and TGF-β, CCL2 as a major chemokine involved in monocyte recruitment, *ERK1*/*2* and *STAT5* signalling pathways activated in Kupffer cells, EPO-R expression and its downstream effects on macrophages [41]. These molecules promote a pro-inflammatory microenvironment and the development of hepatocellular carcinoma. In models of liver injury, NE and epinephrine stimulate NF-κB signalling and collagen production, exacerbating damage [40]. Both the parasympathetic and orthosympathetic systems interact with immune cells such as KCs, dendritic cells, and T cells, suggesting a neuro-immune interaction [5]. Clinical studies show that non-selective beta-blocking drugs reduce markers of systemic inflammation and infections in decompensated cirrhosis, due to lower portal pressure. It reduces bacterial translocation and systemic inflammation, and carvedilol may even exert anti-fibrotic actions (directly on stellate cells and by improving intrahepatic vascular tone) [42,43]

### 2.3. Sensory Innervation

In regions of infection or injury, the activation of peripheral immune cells and the release of cytokines and other inflammatory molecules influence sensory neurons, either triggering or altering signals sent to the spinal cord and brain [5,44]. Sensory innervation is also present in the liver and appears to contribute to regenerative processes, particularly following partial hepatectomy (PH), where it may promote hepatocyte proliferation. Calcitonin gene-related peptide (CGRP), a neuropeptide released from sensory nerve fibres, plays a crucial role in liver regeneration. CGRP acts through its receptor, composed of calcitonin receptor-like receptor (CLR) and receptor activity-modifying protein 1 (RAMP1) [45]. CGRP treatment in liver tissue cultures (both mouse and human) increased Yes-associated protein (YAP) expression and reduced its inhibitory phosphorylation, thereby promoting hepatocyte proliferation [46].

In mice lacking RAMP1, liver regeneration following PH was significantly impaired. These mice showed reduced hepatocyte proliferation and lower expression of YAP/TAZ, key transcriptional coactivators involved in cell cycle regulation and tissue growth [46]. Additional studies confirmed that RAMP1 signalling is essential not only for hepatocyte proliferation but also for angiogenesis, another critical component of liver regeneration. RAMP1-deficient mice had reduced levels of VEGF-C, VEGF-D, and VEGFR3, leading to impaired vascular remodelling post-PH [47].

**Figure 2:**
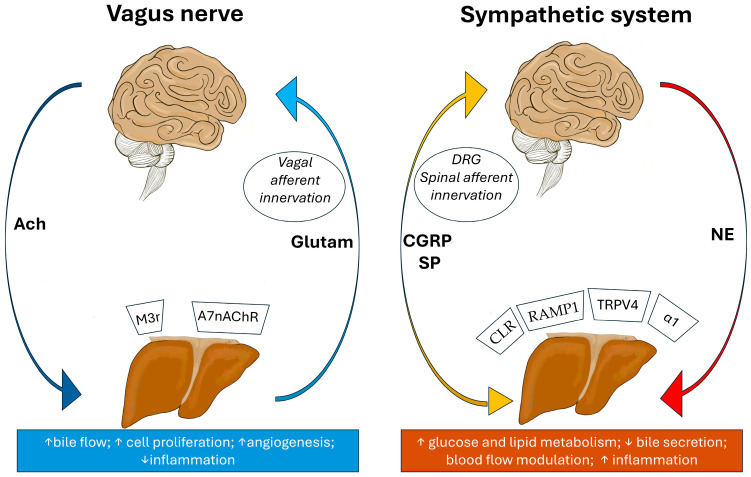
Brain–liver axis illustrating bidirectional neural communication. **Left panel**: Parasympathetic (vagal) pathways. Descending efferents release acetylcholine (ACh) to promote bile flow, hepatocyte proliferation, angiogenesis, and reduce inflammation via receptors such as M3, α7nAChR. Ascending vagal afferents transmit sensory signals to the brainstem primarily via glutamate and do not significantly release CGRP or Substance P in the liver. **Right panel**: Sympathetic pathways. Descending efferents release norepinephrine (NE) to enhance glucose and lipid metabolism, inhibit bile secretion, modulate blood flow, and increase inflammation through α1-adrenergic and TRPV4-mediated mechanisms. Ascending spinal afferents, originating from dorsal root ganglia, have sensory function, but also release neuropeptides such as CGRP and Substance P at their peripheral terminals in the liver, contributing to neurogenic inflammation, vascular modulation, and regeneration via CLR and RAMP1. These circuits form a dynamic neuroimmune–neurovascular interface critical for hepatic homeostasis and disease progression (used Infinite Painter, v. 7.1.18, by M.V.).

This difference may explain variations in liver regeneration and disease progression between species, as well as challenges in studying sensory liver innervation. The limited understanding of hepatic sensory innervation stems from challenges in the histological identification of thin nerve fibres [6], overlapping staining with connective tissues, and technical constraints in electrophysiological recordings, which hinder functional characterisation [48,49]. A part of the sensory fibres is dependent on the neurons of the dorsal root ganglion. They are involved in conveying sensory information like pain, with neuropeptides like CGRP being important markers for these fibres. The calcitonin receptor-like receptor CLR, in complex with RAMP1, is expressed in hepatic vascular compartments and immune cells, where it mediates CGRP signalling. This pathway contributes to angiogenesis, vascular tone regulation, and anti-inflammatory effects, particularly during liver regeneration. While CGRP and Substance P are abundant in spinal afferents, vagal afferents primarily use glutamate as their neurotransmitter for ascending signalling to the nucleus tractus solitarius. Vagal efferents exert cholinergic control through acetylcholine acting on muscarinic (M3) and nicotinic (α7nAChR) receptors, promoting bile flow, hepatocyte proliferation, and immune tolerance. These distinct neurochemical profiles underscore the complementary roles of parasympathetic and sympathetic circuits in the brain–liver axis (Figure 2) [49].

### 2.4. Thyroid Hormone (T3) and Hepatic Neural Influence

The role of thyroid hormones, particularly triiodothyronine (T3), adds another layer of complexity: T3 stimulates hepatocyte DNA synthesis without inducing necrosis and inhibits cholangiocyte proliferation [6,50]. While the direct effects of T3 on parasympathetic nerve endings in the liver remain unclear, it is known to promote the differentiation of oval cells into small hepatocytes, contributing to hepatic hyperplasia [6,51,52].

Some studies suggest that under conditions such as bile duct ligation (BDL), T3 may suppress cholangiocyte proliferation, potentially altering the balance of neural and hormonal signals during liver regeneration [53,54]. This cholangiocyte suppression is mediated through a PLC/IP3/Ca^2+^-dependent pathway, which leads to decreased phosphorylation of Src and *ERK1*/*2*, key regulators of cell proliferation [54]. T3 treatment lowered PCNA expression, a marker of cell proliferation, in isolated cholangiocytes. The inhibitory effect was reversed by blocking PLC or chelating intracellular calcium, confirming the pathway’s involvement [55]. These findings suggest that T3 modulates hepatocyte biliary growth and may influence the balance of regenerative signals during liver injury and repair, particularly in PH and cholestatic conditions like BDL.

A 2022 study by de Assis et al. showed that T3 supplementation rewires liver diurnal transcriptome rhythms, indicating that T3 influences liver physiology in a time-of-day-dependent manner. This suggests a potential interaction between thyroid hormone signalling and neural circadian inputs, as liver clocks are synchronised by neuronal and hormonal signals from the central nervous system [56].

Other studies have demonstrated that T3 stimulates hepatocyte proliferation via the Wnt/β-catenin signalling pathway, mediated by thyroid hormone receptor β (TRβ). This pathway is independent of DNA binding and may involve non-genomic signalling mechanisms, which are often linked to membrane-associated receptors and neural modulation [51]. Moreover, a 2020 review emphasised that thyroid hormones regulate hepatic metabolism and homeostasis through both genomic and non-genomic pathways. Importantly, TRβ is the predominant receptor in the liver, and its activation influences metabolic and vascular tone—functions that are also modulated by autonomic innervation, suggesting a convergent regulatory role [57]. Densitometric assessment of the innervation within the portal space areas indicated a role for noradrenergic fibres in the vascular control during the T3-induced direct hyperplasia rat model, suggesting that under T3 stimulation, the sympathetic nerve fibres are critical in the control of vascular tone [6].

### 2.5. Neurovascular Interactions in the Liver

The liver’s vasculature is subject to neural control, which influences hepatic blood flow and sinusoidal endothelial function [32]. Sympathetic nerves can induce vasoconstriction, reducing blood flow, while parasympathetic nerves promote vasodilation [1,32]. This neural regulation is critical in conditions such as portal hypertension, where altered vascular tone contributes to disease pathology [6].

Neurovascular interactions also involve crosstalk between nerves and hepatic stellate cells, which play a key role in liver fibrosis. Hepatic stellate cells are considered pericytes of the liver in the space between parenchymal cells and sinusoidal endothelial cells of the hepatic lobule [58]. Neural signals can modulate the activation and function of these cells, influencing the progression of fibrotic diseases [1].

Sympathetic nervous system activation leads to contraction of the hepatic artery and reduced permeability of the sinusoidal wall, resulting in decreased hepatic blood flow. The hepatic sinusoids, lined by sinusoidal endothelial cells (SECs) and surrounded by hepatic stellate cells, serve as key sites for blood filtration between the portal and systemic circulation [1]. Efferent hepatic nerve fibres are thought to terminate near HSCs along the sinusoidal wall, where they regulate sinusoidal constriction. Sympathetic neurotransmitters such as adrenaline and substance P induce sinusoidal contraction, while parasympathetic signals, primarily acetylcholine and vasoactive intestinal peptide (VIP), promote relaxation. This neurovascular regulation is especially important during haemorrhage, where sinusoidal constriction helps reduce hepatic blood flow and maintain systemic blood volume [1,58,59]. There are plausible neural–coagulation cross-talk pathways through which hepatic innervation can modulate fibrogenic processes initiated by thrombophilia (e.g., portal vein thrombosis, microthrombosis, and parenchymal extinction). Direct human evidence that liver nerves initiate thrombosis is limited but several well-supported mechanisms show how coagulation proteases and autonomic signals converge on hepatic stellate cells (HSCs), sinusoidal endothelium, and intrahepatic vascular tone, potentially amplifying fibrosis once a thrombotic trigger is present [1,60].

Neurovascular communication plays a fundamental role in the brain-liver axis in a bidirectional way. Altered bile acid composition due to liver dysfunction affects blood–brain barrier permeability, neuroinflammation, and synaptic function, linking liver health to neurological disorders. Bile acids may serve as signalling molecules in the gut–liver–brain axis, influencing diseases like Alzheimer’s and Parkinson’s [61]. As such, the liver, acts as an endocrine organ, releasing hepatokines and metabolites that influence brain metabolism and neuroprotection [62]. Therefore, the dynamic communication between the brain and liver is of critical importance, as each organ profoundly influences the other. Neural innervation regulates hepatic vascular tone and blood flow, while the liver’s vascular health and metabolic status directly impact cerebral function and systemic homeostasis.

The endothelial cells of hepatic sinusoids produce, among other molecules, ephrins [63]. This may be one of the important molecules for the liver’s neuro-vascular relationship because of its actions: indeed, Ephs and ephrins regulate axon pathfinding by eliciting repulsive or attractive cues [64].

While not fully delineated in the liver, recent studies revealed that, in general, nerves and blood vessels develop in parallel, forming tightly aligned networks essential for organogenesis, tissue repair, and homeostasis [65].

Such neurovascular co-dependence within the liver may assume an important significance because, first, neurovascular proximity facilitates coordinated signalling between endothelial cells and nerve fibres; second, disruption of these interactions contributes to disease progression, including fibrosis and cancer [66,67,68]. Third, angiocrine factors released by endothelial cells and neurotransmitters from autonomic nerves regulate liver function and regeneration [65].

The peribiliary plexus (PBP) and hepatic innervation are two key components of the liver’s regulatory system, especially in relation to the biliary tree. The PBP is a dense capillary network that surrounds the intrahepatic bile ducts, primarily derived from branches of the hepatic artery (Figure 1).

PBP’s main roles include providing oxygen and nutrients to the biliary epithelium (cholangiocytes) and facilitating the transport of molecules involved in bile modification [69]. PBP also acts as a selective barrier and may participate in immune surveillance. As already seen, neural signals influence cholangiocyte activity, which is supported by PBP’s vascular supply. Nerve fibres run alongside such blood vessels, including those forming the PBP [70]. Therefore, neurotransmitters (e.g., norepinephrine, acetylcholine) could modulate vascular tone and blood flow in the PBP [71]. In conditions like cholestasis or bile duct ligation, cholangiocytes proliferate and secrete VEGF, promoting expansion of the PBP [69], and it has been shown that such biliary proliferation is paralleled by an increase in innervation [6].

Beyond haemodynamic and metabolic inputs, the functional output of hepatic neural circuits is critically shaped by the availability and turnover of neurotransmitters acting on intrahepatic targets and, in some instances, on nerve terminals themselves. Among sympathetic mediators, catecholamines (noradrenaline/adrenaline) drive glucose and lipid mobilisation and modulate inflammatory and fibrogenic pathways via adrenergic receptors on hepatocytes, Kupffer cells, and hepatic stellate cells; experimental and clinical contexts demonstrate that changes in catecholamine levels translate into altered hepatic sympathetic tone and immune activation [72,73]. Neurotransmitter quantity is not a passive readout but a primary determinant of hepatic neural and vascular tone and downstream pathophysiology, particularly in steatotic and cholestatic contexts. The availability and balance of neurotransmitters (catecholamines, neuropeptides, serotonin, acetylcholine, and purines) dynamically tune hepatic autonomic and sensory signalling, thereby shaping metabolic, inflammatory, fibrogenic, and regenerative responses [72,73].

While chronic liver disease offers more extensive evidence for neuroimmune-neurovascular regulation, emerging studies in acute liver injury, notably the vagus–macrophage–hepatocyte (*FoxM1*) and CGRP–RAMP1–YAP/TAZ pathways, indicate that neural inputs also orchestrate early immune and vascular responses, albeit with fewer human datasets to date. Accumulating evidence indicates that acute liver injury (ALI) rapidly recruits the neuroimmune–neurovascular interface. Autonomic efferents, especially vagal cholinergic signals, modulate Kupffer cell cytokine output and microcirculatory tone, thereby shaping the inflammatory set point and priming hepatocyte cell-cycle re-entry. Mechanistic studies demonstrate a vagus–macrophage–hepatocyte axis that drives *FoxM1*-dependent regeneration after injury [74]. In parallel, sensory neuropeptide pathways (e.g., CGRP acting via the CLR/RAMP1 complex) couple afferent fibres to YAP/TAZ activity, endothelial remodelling, and angiogenesis in the regenerating liver, underscoring that neural cues are integral to the restoration of perfusion and parenchyma following ALI [46]. Collectively, these observations support the view that neural inputs orchestrate early immune and vascular responses in acute conditions and may represent actionable targets for bioelectronic or pharmacological neuromodulation.

In liver oncology, the neuro-vascular interaction of the liver takes on particular importance. Until recently, the impact of nerves on cancer development, progression, and metastasis has been largely unknown. Sympathetic and parasympathetic nerves influence vascular remodelling via the release of growth factors like VEGF and blood vessel formation, and facilitate tumour progression through immune modulation. In addition, autonomic innervation influences intratumoral nerve density, which correlates with poor prognosis and increased vascular invasion [75].

These data underscore the role of nerve fibres in coordinating vascular growth, immune suppression, and tumour progression. Understanding these interactions opens avenues for targeted neuromodulatory therapies in malignant and non-malignant liver diseases.

## 3. Hepatic Innervation in Specific Diseases

### 3.1. Liver Fibrosis/Cirrhosis

In cirrhosis, hyperammonaemia and other neurotoxins (e.g., bile acids, manganese) induce CNS dysfunction via astrocytic/blood–brain barrier (BBB) pathways and contribute to PNS/ANS injury (length-dependent polyneuropathy, autonomic neuropathy, hepatic myelopathy), with diagnostic implications and therapeutic avenues (lactulose/rifaximin, microbiota-targeting, transplantation in selected cases) [76]. Beyond ammonia, BBB dysfunction (tight-junction loss, aquaporin-4 mislocalisation, pericyte injury, altered transporters) amplifies permeability and neuroinflammation, while bile acids (altered pool/composition in cholestasis) modulate BBB flux, microglial activation, and synaptic function [77].

Sympathetic innervation has been closely linked to the pathophysiology of liver fibrosis and cirrhosis [5,6]. In humans and other mammals, sympathetic fibres target the sinusoids and hepatic stellate cells, which are central to the remodelling of the sinusoidal microenvironment during fibrotic progression. Experimental studies have shown that adrenergic antagonists can attenuate liver fibrosis, suggesting a direct role of sympathetic signalling in modulating extracellular matrix deposition. In liver fibrosis, it has been demonstrated that the α2-AR antagonist mesedin effectively deactivated HSCs and increased the permeability of human liver sinusoidal endothelial cells [78].

In advanced fibrosis and cirrhosis, sympathetic fibres are prominent in the connective septa and portal tracts but are reduced in the parenchyma and absent in cirrhotic nodules [79]. Animal models, particularly those involving carbon tetrachloride (CCl4)-induced liver injury and biliary duct ligation, have demonstrated the involvement of adrenergic pathways in both the progression and potential regression of fibrosis [6,80]. These findings highlight the importance of sympathetic innervation in regulating hepatic stellate cell activity, oval cell proliferation, and systemic inflammatory responses.

Experimental models support the regenerative and fibrogenic role of parasympathetic innervation. In mice, vagotomy impairs the regenerative response of hepatocytes and oval cells following subacute liver injury by disrupting the vagus–macrophage–hepatocyte signalling axis [74].

In chronic liver damage, hepatocyte regeneration is often accompanied by cholangiocyte proliferation within the portal spaces, a process influenced by neuroendocrine signals and the surrounding microenvironment. Ductular reactions (DRs) emerge, characterised by cholangiocyte proliferation in portal spaces. These cells interact with hepatic stellate cells and immune cells, contributing to fibrosis and regeneration [81]. It has been demonstrated in the Sprague Dawley rat model with CCL4-induced fibrosis that an increase in cholinergic nerve fibres develops around these ductal proliferations, and the cholinergic signalling through the α7 nicotinic acetylcholine receptor on Kupffer cells exerts an intrinsic protective role in the cirrhotic liver by modulating the inflammatory pathways [6,31].

Hepatic stellate cells themselves are capable of synthesising acetylcholine and express muscarinic receptors, suggesting that parasympathetic signalling may also influence fibrogenesis, as ACh acts in an autocrine/paracrine manner to regulate HSC proliferation and activation [82].

Hepatitis C virus infection is strongly associated with fibrosis and increased thrombotic risk in hepatic circulation [60]. Increased thrombin formation may activate hepatic stellate cells and promote liver fibrosis, microvascular thrombosis and parenchymal extinction, increasing thrombin exposure in the sinusoidal niche. Thrombin (and FXa) directly activate hepatic stellate cells via PAR-1/-4, inducing contraction and profibrotic gene programs; PAR-1 antagonists and factor-Xa inhibition limit fibrosis in vivo. In parallel, the hepatic sympathetic network modulates HSC biology: catecholamines and NPY from nerves (and HSC autocrine sources) enhance proliferation and collagen synthesis. While neuronal PAR signalling is well described in extrahepatic tissues, direct evidence that thrombin activates intrahepatic nerves in HCV to drive fibrosis is not yet available. Is a thrombin–nerve axis operative in HCV-related fibrosis? We therefore posit a cooperative model in which coagulation-derived PAR signalling and autonomic neurotransmission converge on HSCs and sinusoidal endothelium to amplify fibrogenesis during HCV-related injury [83,84].

### 3.2. MASLD/MASH

The terms Metabolic Dysfunction-Associated Steatotic Liver Disease (MASLD) and Metabolic Dysfunction-Associated Steatohepatitis (MASH) refer to stages of a liver condition related to metabolic dysfunction, and they represent a shift in medical terminology from the older terms NAFLD (non-alcoholic fatty liver disease) and NASH (Non-Alcoholic Steatohepatitis). MASLD can remain stable or progress to MASH if inflammation and liver cell damage occur.

In MASLD and MASH, sympathetic overactivity is linked to metabolic dysregulation and inflammatory amplification. Neural pathways contribute to the pathogenesis and progression of these conditions [85].

Recent studies demonstrated that endoplasmic reticulum (ER) stress in neurons projecting from the subfornical organ (SFO) to the hypothalamic paraventricular nucleus (PVN) contributes to MASLD by modulating sympathetic nervous system activity. This neural circuit influences hepatic lipid acquisition, and inhibition of ER stress in these neurons reduced liver triglyceride levels and tyrosine hydroxylase (TH, a marker of sympathetic activity), confirming the neurogenic contribution to hepatic steatosis [86].

The impact of liver-projecting vagal sensory neurons on energy balance, hepatic steatosis, and anxiety-like behaviour has been demonstrated in mice under obesogenic conditions [87]. The vagus nerve regulates satiety (vagal-dependent actions of GLP-1) and energy homeostasis. Dysfunction in vagal signalling contributes to obesity and MASLD/MASH progression. Moreover, TGF-β signalling, which is influenced by neural pathways, promotes hepatic inflammation and fibrosis, linking neural dysregulation to liver pathology [88].

Autocrine/paracrine mechanisms of ACh in regulating HSC are implicated in liver fibrosis, especially in conditions like the previously called non-alcoholic steatohepatitis (NASH). Crunkhorn et al. identified autocrine signalling circuits in HSCs as key drivers of fibrosis in NASH. Although their focus was on NTRK3–NTF3 interactions, the concept of HSCs engaging in neurotransmitter-like autocrine loops supports the plausibility of acetylcholine-mediated signalling in these cells [89].

Owaki et al. demonstrated that the autonomic liver–gut neural axis, particularly involving serotonin (5-HT) and its receptor HTR2A, plays a role in NASH: Blocking hepatic nerves reduced intestinal 5-HT, hepatic HTR2A expression, and lipogenic gene activity. This axis is independent of central neural pathways and represents a peripheral neuroendocrine mechanism in NASH pathogenesis [90].

### 3.3. ALD

#### 3.3.1. Central Nervous System (CNS) Injury in AUD/ALD

Alcohol Use Disorder (AUD) is a widespread condition characterised by compulsive alcohol consumption that significantly impairs an individual’s physical health, occupational functioning, and social relationships. The prevalence of AUD rose notably during the COVID-19 pandemic, further intensifying its impact as a public health concern. In Ireland, i.e., AUD accounted for approximately 4% of all deaths between 2008 and 2017, underscoring its substantial societal and healthcare burden [91].

In alcohol-associated liver disease (ALD), the stress axis and sympathetic drive play significant roles in immune activation and liver injury [91]. Neural mechanisms contribute to the inflammatory and fibrotic responses observed in ALD. Chronic alcohol intake activates the hypothalamic–pituitary–adrenal (HPA) axis, increasing cortisol and sympathetic tone. This axis activation promotes Kupffer cell activation, cytokine release, and hepatic stellate cell activation by activating α/β-adrenergic receptors, driving fibrosis [92]. This leads to the increased production of collagen, TGF-β, and IL-6, and activation of NF-κB, promoting a pro-inflammatory and fibrogenic environment. These mechanisms are implicated in both ALD and hepatocellular carcinoma [40]. Alcohol metabolism generates reactive oxygen species (ROS) and acetaldehyde, which sensitise the liver to immune-mediated damage [93]. Alcohol produces dose- and pattern-dependent neurotoxicity through oxidative stress (including CYP2E1-mediated ROS/RNS), neuroinflammation, mitochondrial/ER stress, and dysregulated autophagy/mitophagy, ultimately impairing neuronal survival and plasticity. These pathways are consistently reported across acute and chronic exposure models and human studies [94,95]. Functionally and structurally, alcohol impacts hippocampal neurogenesis (memory), prefrontal cortex (executive control), and cerebellum (motor coordination). Hippocampal atrophy and reduced neurogenesis correlate with cognitive decline and blackouts, whereas cerebellar degeneration contributes to chronic ataxia [96]. These CNS mechanisms integrate with liver disease trajectories via the liver–brain axis, where inflammatory mediators, endotoxins, and altered bile acid profiles contribute to neuroinflammation and cognitive impairment in chronic liver disease [5].

#### 3.3.2. Peripheral and Autonomic Nervous System (PNS/ANS) Injury in AUD/ALD

Alcohol related peripheral neuropathy (ALN) is common in chronic heavy drinkers, typically presenting as a length-dependent, predominantly sensory axonal neuropathy; pooled prevalence approaches ~46% when confirmed by nerve conduction studies [97]. Recent studies have increasingly highlighted the vagus nerve’s role in alcohol consumption and alcohol use disorder, particularly through its influence on neuroimmune regulation, cognitive control, and inflammatory responses. This pathway is being explored as a non-invasive therapeutic target for liver diseases, including ALD. Although the role of the Dorsal Vagal Complex (DVC) in AUD is not well characterised, several studies suggest that the DVC may be a critical area of interest for future AUD therapies. Since the DVC is a primary input region of the vagus and other peripheral nerves, regulating autonomic output, the DVC and its various neurotransmitter systems can be examined and/or modulated, either directly or indirectly, in clinical and preclinical studies [98]. This enables several future translational studies that can impact AUD research. In a clinical context, it has been demonstrated that the Roux-en-Y gastric bypass surgery (RYGB) can lead to increased alcohol use and AUD in humans, even in those individuals without a history of significant alcohol use, with similar findings in rodent models. While the exact underlying mechanism remains unknown, pre-clinical studies of RYGB point to neuronal plasticity occurring in the DVC following the removal of gastric vagal afferents [67,68,98]. Mechanistically, ALN reflects direct ethanol/acetaldehyde neurotoxicity, oxidative stress, and nutritional deficiencies, especially thiamine, with contributions from metabolic dysregulation and liver dysfunction; small fibre (including autonomic) involvement is frequent [97].

Autonomic dysfunction (cardiovascular reflex test abnormalities) affects ~16–73% of chronic alcohol abusers and relates strongly to lifetime ethanol dose; abstinence is the main intervention associated with improvement in autonomic testing [99]. Therapeutic strategies centre on sustained abstinence, nutritional support, and B vitamin supplementation (notably thiamine); while evidence is limited, these measures are recommended to reduce progression and symptom burden [97,99].

### 3.4. Autoimmune Liver Diseases

In autoimmune liver diseases such as autoimmune hepatitis and primary biliary cholangitis, neural modulation of immune tolerance is a critical factor. Understanding the role of liver innervation in these conditions may reveal new therapeutic targets for modulating immune responses and disease progression.

Autoimmune hepatitis (AIH) is a chronic inflammatory liver disease characterised by interface hepatitis, lobular necroinflammation, hypergammaglobulinemia, and the presence of circulating autoantibodies [100]. Traditionally considered a disorder of dysregulated adaptive immunity, AIH now offers a clinically relevant framework for examining how liver innervation intersects with immune regulation and metabolic control. Emerging evidence suggests that the liver’s neural network—comprising sympathetic, parasympathetic, and sensory fibres—not only influences local immune tolerance but also contributes to systemic manifestations and disease progression [31,61,62,91].

Sympathetic and parasympathetic neural circuits coordinate metabolic and detoxification processes and also elicit immunomodulatory effects [8]. In the context of AIH, disrupted neurotransmitter-mediated signalling—particularly involving norepinephrine effects on dendritic cells and T lymphocytes—may compromise immune tolerance and amplify liver injury [5,66,101].

Abnormal neuroimmune signalling may also underlie several systemic symptoms frequently reported in AIH. Fatigue and pruritus, for example, may reflect dysfunctional communication between the liver and the CNS. In cholestasis, bile acids activate hepatic sensory neurons expressing TGR5 and MRGPRs, triggering non-histaminergic pruritus via spinal and central neural pathways [102,103]. This may reframe pruritus not merely as a dermatologic symptom but as a neurologically mediated phenomenon rooted in hepatic sensory dysregulation.

The gut–liver–brain axis provides further insight into this neuro-visceral interplay. Vagal afferents act as conduits for immune and metabolic signals, relaying information from the gut and liver to central nuclei [88]. Vagal nerve stimulation (VNS) has demonstrated anti-inflammatory effects and improved insulin sensitivity in models of metabolic-associated steatohepatitis, a condition that shares immunometabolic pathways with AIH. These findings underscore the therapeutic potential of neuromodulation in immune-mediated liver diseases [104].

Sympathetic innervation also contributes to hepatic metabolic regulation through CNS-liver signalling. Fibroblast growth factor 21 (FGF21), for example, acts via both central and peripheral mechanisms to reverse steatohepatitis, with sympathetic input modulating very-low-density lipoprotein (VLDL) secretion [105]. Autoimmune markers may appear in MASH, complicating diagnosis, so biopsy and digital pathology are essential for accurate differentiation [106]. Given the mechanistic overlap between MASH and AIH [107], these neuroendocrine pathways may inform novel therapeutic strategies in autoimmunity-driven liver injury.

Autonomic dysfunction may also affect outcomes following liver transplantation. Altered sympathetic and parasympathetic signalling has been linked to impaired lipid metabolism in the post-transplant setting, with potential implications for graft function in patients with MASH or autoimmune liver diseases [108]. These observations highlight the importance of autonomic integrity in maintaining immunometabolic stability in the transplanted liver.

An additional layer of neuro-metabolic regulation involves the dorsal motor nucleus of the vagus (DMV), which controls jejunal microvilli and intestinal lipid absorption. Altered DMV activity can influence hepatic lipid load and contribute to steatotic injury [109,110]. These insights could provide a broader understanding of autoimmune liver diseases (AILDs), including AIH, as disorders at the intersection of immunity, metabolism, and neural regulation. In this model, neural circuits modulate not only local immune activation but also systemic metabolic balance and clinical manifestations.

Clinical observations further suggest that environmental and viral triggers play a role in shaping disease onset and progression. SARS-CoV-2 infection, for instance, has been associated with de novo AIH, potentially through mechanisms such as molecular mimicry or bystander activation [111]. At the molecular level, tolerogenic molecules such as HLA-G may affect immune homeostasis in AIH. Elevated levels of soluble HLA-G in type 1 AIH suggest a compensatory immunosuppressive response, though persistent disease activity points to an incomplete restoration of immune tolerance [112].

Despite growing interest in non-invasive diagnostics, liver histopathology remains a cornerstone for diagnosing, staging, and managing AIH [112,113]. A multidisciplinary approach—grounded in collaboration between clinicians and pathologists—enhances the interpretive value of biopsy, especially in complex or overlapping syndromes [114].

Altogether, these findings contribute to a paradigm shift in our understanding of AIH: from a purely immunological disease to a multifaceted neuroimmune-metabolic disorder, with implications for both pathophysiology and therapeutic innovation.

### 3.5. Hepatic Malignancies

The nervous system interacts with the liver’s immune environment in ways that are increasingly recognised as critical in the development and progression of liver cancer [75,115]. The concept of a “neuroimmune axis” has emerged, highlighting how neural signals can shape the tumour microenvironment. Through the release of catecholamines, sympathetic nerves can alter glucose and lipid metabolism, promote inflammation, and contribute to disease progression.

In contrast, parasympathetic innervation via the vagus nerve exerts anti-inflammatory effects and supports tissue regeneration. Acetylcholine released from vagal terminals interacts with immune cells, particularly Kupffer cells, to suppress pro-inflammatory cytokine production and maintain immune homeostasis. Intriguingly, while vagal signalling exerts protective anti-inflammatory effects and may be protective in the context of inflammation-driven malignancy, it may also dampen anti-tumour immunity and facilitate tumorigenesis and tumour progression. For instance, ACh receptor agonists have been shown to increase tumour burden and reduced CD8+TNFα+ subsets [116]. These findings emphasise the need for context-specific neuromodulatory approaches, especially considering the liver as a common site of metastasis.

In hepatocellular carcinoma (HCC), sympathetic nerves may also promote tumour growth and metastasis by modulating immune cell infiltration and function, contributing to CD8+ T cell exhaustion, through the β1-adrenergic receptor [117]. Conversely, interventions that disrupt neural input, such as neurolysis, are being explored as potential therapeutic strategies to alter the tumour milieu and improve treatment outcomes [116,118].

This evolving understanding of the liver’s neuroanatomy and its functional implications underscores the importance of integrating neuroscience into hepatology (as well as for other viscera). The nervous system is emerging as an active participant in liver health and disease, influencing everything from metabolic regulation to immune surveillance, immune tolerance and inflammation and depending on context, contributing to or limiting the progression of non-malignant and malignant disease (Table 1).

## 4. Emerging Research and Therapeutic Approaches

Emerging research models in the field of liver innervation and regeneration are changing perspectives in therapeutic approaches that aim to leverage the neural regulation of liver functions to treat liver diseases.

### 4.1. Vagus Nerve Stimulation (VNS)

Neuromodulatory drugs, including β-blockers and cholinergic agents, are being explored for their potential to influence liver function and disease progression. Alongside these approaches, bioelectronic medicine, which involves using electronic devices to modulate neural activity, represents a future direction for therapeutic intervention [104,119,120]. Vagus nerve stimulation (VNS), as mentioned above, has shown promise in modulating inflammation and improving outcomes in various liver conditions. These approaches hold the potential to provide personalised and targeted treatments for liver disease via the modulation of neural pathways.

Next-generation bioelectronic medicine includes closed-loop systems that monitor physiological signals in real-time and adjust stimulation accordingly. These systems can dynamically modulate autonomic nervous system activity, including hepatic sympathetic and parasympathetic pathways [121]. Applications include sepsis, chronic inflammation, and potentially metabolic liver diseases like MASLD and NASH.

Vagal stimulation enhances DNA synthesis and hepatocyte proliferation, partly through the release of interleukin-6 from macrophages and activation of the transcription factor *FoxM1* [74]. It must be remembered that while vagal stimulation is protective in inflammation, it may suppress anti-tumour immunity in certain hepatic cancers. Therefore, responses may differ based on disease stage, comorbidities, and neural and liver anatomy. Integration with existing therapies: combining neuromodulation with pharmacological or lifestyle interventions will be key.

Vagal stimulation holds significant promise for personalised and non-pharmacological treatment of liver diseases. Ongoing research and technological innovation will likely expand its clinical applications, making it a cornerstone of next-generation hepatology care.

### 4.2. Liver Organoids

Liver organoids are three-dimensional structures derived from pluripotent stem cells (PSCs) or adult stem cells that have emerged as powerful and versatile platforms for disease modelling [115] due to their capacity to recapitulate the structural and functional complexity of the human liver. Organoid-based systems enable detailed investigation of liver disease initiation, progression, and therapeutic response, thereby providing a translational bridge toward personalised medicine [122], and this could be important to model neural–liver interactions and test neuromodulatory therapies.

A major advantage of liver organoids lies in their physiological relevance. In contrast to conventional two-dimensional culture systems and animal models, liver organoids recreate the native hepatic microenvironment, capturing the cellular heterogeneity and spatial organisation characteristic of the human liver [123]. This fidelity to in vivo conditions is particularly valuable for modelling chronic liver diseases, in which tissue remodelling, inflammation, and multicellular interactions critically shape disease onset and evolution. Moreover, liver organoids can be genetically engineered to introduce disease-specific mutations, establishing a robust and controllable platform for studying genetic liver disorders and elucidating their underlying molecular mechanisms.

Patient-derived liver organoids have emerged as transformative platforms for advancing precision hepatology, enabling individualised drug screening, mutation analysis, and prediction of therapeutic response. By faithfully capturing the genetic, metabolic, and phenotypic heterogeneity of patient tissues, these ex vivo composite models facilitate the development of tailored therapeutic strategies that align with an individual’s unique molecular and physiological profile [124]. This capability holds particular promise in addressing interpatient variability in drug metabolism and disease progression, long-standing challenges that have limited the efficacy of conventional, population-based therapeutic approaches.

Recent advances, comprehensively reviewed by Fang et al. [125], highlighted the broad utility of human hepatobiliary organoids in modelling diverse hepatic and biliary diseases, including viral hepatitis, liver fibrosis, hepatocellular carcinoma, and cholangiopathies such as biliary tract cancers. These organoid systems have proven valuable not only for drug toxicity testing and high-throughput pharmacological screening but also as experimental platforms for precision medicine, given their ability to maintain the histological architecture, cellular diversity, and physiological functions of native liver tissue.

Moreover, patient-derived organoids serve as a critical translational bridge between two-dimensional in vitro cultures and in vivo animal models, capturing complex multicellular interactions and microenvironmental cues that are otherwise lost in reductionist systems. Although technology remains in its developmental phase, remarkable progress has been made in generating multicellular liver organoids that incorporate hepatocytes, cholangiocytes, stellate cells, endothelial cells, and immune components in physiologically relevant proportions and spatial arrangements [123,126]. Such sophisticated models increasingly emulate the structural organisation and functional integration of the human liver, offering unprecedented opportunities to dissect the molecular mechanisms underlying liver disease and to test personalised therapeutic interventions.

As the field advances, patient-specific liver organoids are expected to become integral components of precision medicine frameworks, complementing in silico patient digital twins and clinical datasets to refine diagnosis, optimise therapeutic regimens, and predict patient outcomes. Collectively, these developments signify a paradigm shift in hepatology—from generalised treatment strategies toward mechanistically informed, patient-tailored interventions that hold the potential to improve efficacy, safety, and long-term clinical outcomes.

Despite their considerable promise, current liver organoid models continue to face important limitations. Although they successfully recapitulate key pathological and physiological features of liver disease, their hepatocyte-specific functions remain less mature than those of primary hepatocytes, exhibiting characteristics reminiscent of progenitor- or foetal-like states. Consequently, the optimisation of culture conditions, biochemical cues, and differentiation protocols to drive hepatocyte maturation represents a critical prerequisite for enhancing their translational relevance and predictive fidelity in disease modelling and therapeutic testing.

### 4.3. Digital Twins

Digital twins are advanced computational models that leverage artificial intelligence (AI) to emulate the structural and functional properties of physical systems [127]. In liver research, digital twins enable the simulation of hepatic conditions and hold the potential to model neural–liver interactions for predicting therapeutic responses. By integrating multimodal data, including imaging and electrophysiological and multi-omics datasets, these models offer a comprehensive and dynamic representation of liver function, encompassing its neural innervation.

A valuable example of the above concept has been expressed by the virtual hepatic lobule [128], the foundational and functional unit of the living liver, a multiscale virtual twin of the human liver. This model integrates hepatic blood flow dynamics with a clinically informed framework of acetaminophen-induced injury, enabling the prediction of patient-specific hepatocellular damage patterns. By incorporating metabolic zonation, those simulations closely reproduced clinically observed zonal hepatotoxicity. These findings demonstrated the predictive capability of the virtual hepatic lobule and established a critical step toward the realisation of a comprehensive human liver virtual twin with physiological fidelity across spatial and temporal scales, with future research possibly focused on refining the hepatocyte regeneration driven by mitogens and nutrients. The literature underscores that additional efforts will provide a model for the retrograde flow of hepatic lymph within the space of Disse and to integrate spatially resolved hepatic gene expression data governing xenobiotic transport and metabolism. Such integration will enable genetic parameterisation of the model and support the exploration of pharmacogenomic determinants of drug-induced liver injury (DILI).

Another study inherent to DILI presented the development of a digital twin of the human liver, constructed through the integration of mechanistic knowledge derived from studies of hepatic physiology, pathology, and pharmacology within a mathematical framework based on a differential equation-based framework of hepatic dynamics. The digital twin accurately reproduced normal liver function, the progression of disease, and the effects of therapeutic interventions. Furthermore, coupling the model with experimental data empowered mechanistic insights into DILI. Remarkably, the methodology outlined here is broadly applicable, offering a general framework that can be extended to other organs and biological systems to advance safer and more efficient drug development [129].

Moreover, a new paradigm in drug development and therapeutic strategy design is urgently needed, particularly for complex and heterogeneous diseases such as metabolic dysfunction-associated steatotic liver disease (MASLD). The pronounced heterogeneity among MASLD patients-driven by genetic variation, comorbidities, gut microbiome composition, lifestyle, environmental exposures, and demographic factors, results in diverse clinical presentations and outcomes. Conventional drug development approaches have achieved only limited success in such contexts: only a subset of patients respond to available therapies, therapeutic response prediction remains elusive, and the high cost of novel therapeutics further constrains clinical translation. Emerging patient digital twins (PDTs) offer a transformative approach to this challenge. Complementing these in silico models, patient biomimetic twins (PBTs)—derived from patient-specific organoids or induced pluripotent stem cells (iPSCs) differentiated into organ-relevant cell types—serve as experimental avatars that recapitulate patient-specific pathophysiological mechanisms. These biomimetic systems enable the validation and refinement of PDT-based predictions under controlled experimental conditions. The integration of PDTs and PBTs represents a promising frontier in precision medicine, bridging computational modelling with experimental biology to personalise therapy development and evaluation. While substantial challenges remain, including data standardisation, model interpretability, and translational validation, the convergence of these digital and biomimetic platforms has the potential to redefine the paradigm of individualised therapeutic discovery and optimisation for heterogeneous diseases such as MASLD [130].

An alternative virtual approach has been introduced by DigiLoCs [131], a digital twin of a liver-on-chip platform that accurately replicates the hepatic clearance functionality of the human liver. This model integrates biological, physicochemical, and hardware-specific data within a mechanistic framework based on ordinary differential equations. This setting provided two major advantages: (1) improved prediction of intrinsic hepatic clearance compared with conventional models, and (2) enhanced mechanistic interpretability through the explicit representation of physiological parameters governing drug disposition. To demonstrate the translational relevance of the framework, propranolol was used as a proof-of-concept compound, and physiologically based pharmacokinetic modelling was employed to extrapolate in vitro clearance predictions to the human setting. The ultimate goal gave rise to digital twins capable of predicting clinical outcomes more accurately, thereby reducing the time, cost, and patient burden in drug development. The DigiLoCs software captures complex liver-on-chip biological behaviour, combining mechanistic modelling of clearance, permeability and partitioning processes, hardware-specific parameters from the liver-on-chip system, and compound-specific characteristics influencing hepatic disposition. Unlike conventional models that aggregate all elimination processes into a single clearance term, DigiLoCs differentiate between active biological processes (e.g., metabolism) and passive physicochemical processes (e.g., permeability and partitioning). Finally, DigiLoCs depicted a mechanistically transparent and quantitatively robust mapping between in vitro and in silico systems, enabling the disentanglement of active and passive processes and improving the predictive accuracy of hepatic clearance across scales.

A very sophisticated application of digital twins has been tailored to the extraordinary regenerative capacity of the liver following partial resection that underpins the success of living donor liver transplantation (LDLT). However, substantial donor heterogeneity influences the rate and extent of post-surgical recovery, underscoring the need for individualised monitoring strategies. With the global rise in liver diseases, advancing safer transplantation protocols and optimised donor care have become increasingly critical. Current clinical biomarkers offer only limited, time-specific insights into recovery, hindering the prediction of long-term regenerative outcomes. Liver mass restoration after partial hepatectomy relies on precisely orchestrated hepatocyte proliferation. To elucidate this process, Halder et al. [132] analysed longitudinal blood-derived gene expression profiles through a deep learning-integrated mechanistic modelling framework. The identification of distinct transcriptional signatures associated with regeneration was paramount for the incorporation into a Personalised Progressive Mechanistic Digital Twin, a virtual liver model capable of predicting donor-specific recovery trajectories. The novelty of this work lies in the creation of a hybrid modelling architecture that seamlessly integrates mechanistic mathematical modelling with deep learning-based autoencoders to construct an interpretable digital twin of liver regeneration and represents the first digital twin trained on longitudinal transcriptomic data from LDLT donors to model hepatic regeneration. This established a new paradigm in interpretable, personalised predictive modelling for regenerative medicine.

Finally, the critical importance of verification, validation, and uncertainty quantification [133] should be highlighted in establishing trustworthy and ethically grounded digital twins for precision medicine. Beyond ensuring predictive accuracy and scientific rigour, effective validation integration must also protect individual privacy and data security, addressing ethical considerations at every stage of model development and deployment. By embracing these principles, the biomedical community can accelerate the safe and equitable adoption of digital twins, transforming personalised, data-driven healthcare from an aspirational goal into a clinical reality.

Taken together, these insights illuminate a pivotal moment in biomedical science, where emerging digital technologies and AI are redefining the boundaries of both basic and clinical research. If guided by robust scientific principles and applied with ethical responsibility, this digital transformation holds the potential to revolutionise the research-to-clinic continuum, reducing resource and energy demands, accelerating the pace of drug discovery, and reshaping therapeutic pathways through the adoption of less invasive, data-driven diagnostic strategies. The responsible integration of these tools marks not merely an evolution in methodology, but a paradigm shift toward a more intelligent, efficient, and human-centred future for medicine.

### 4.4. Biomaterials for Liver Tissue Engineering: Enabling Models and Therapies Targeting the Neuroimmune-Vascular Interface

The biomaterials used in tissue engineering perhaps expand the horizons for both the basic study of liver regeneration and the development of personalised therapies.

The liver’s dense innervation intricately coordinates vascular tone, metabolic regulation, and immune surveillance, critically influencing hepatic function and disease pathogenesis. However, current biomaterial-based liver tissue engineering (TE) strategies often neglect this neuroimmune–vascular interface, limiting their capacity to model and modulate the integrated pathways governing inflammation, fibrosis, and vascular dysfunction in liver disease. To address these limitations, the field is focusing on the development of next-generation biomaterials that are ideally biomimetic, bioresponsive, and structurally faithful to the liver’s complex anatomical architecture, while actively incorporating neuroimmune and vascular components to enhance physiological relevance and therapeutic potential.

First, designing biomaterials with biomimetic physicochemical properties is a critical step toward accurately modelling the mechanical microenvironment that governs nerve–vascular–immune crosstalk in the liver. The compliant, viscoelastic nature of the liver (~0.5–1 kPa) modulates sinusoidal endothelial alignment, hepatic stellate cell (HSC) activation, and immune cell trafficking while influencing nerve fibre ingrowth and function. Efforts have focused on engineering hydrogels with tunable stiffness, such as GelMA, to support hepatocyte functionality while preserving endothelial integrity [134,135,136]. Decellularised liver ECM scaffolds represent another type of engineered biomaterial that preserves liver-specific biochemical signals, enhancing hepatocyte albumin production, cytochrome P450 activity, and endothelial nitric oxide synthesis [137,138,139]. While effective in maintaining liver-specific signals, these first-generation isotropic biomaterials often lack the mechanical and topographical cues required for neurite outgrowth, which is essential for modelling liver innervation and its regulatory roles in hepatic function.

Although largely underexplored in liver TE, anisotropic scaffolds and gradient-stiffness hydrogels have been effectively applied in peripheral nerve repair and spinal cord regeneration to guide neurite extension, Schwann cell alignment, and coordinated vascularisation [140,141]. Adapting aligned and gradient-stiffness scaffolds from these fields could similarly advance neurovascular integration in liver TE, enabling improved models and neuromodulatory therapies for liver disease.

Second, bioresponsive biomaterials integrating advanced drug delivery and gene therapy strategies are actively being explored to modulate the immune environment during liver regeneration and disease modelling, although their application in targeting the neuroimmune axis within liver TE remains underexplored [142,143,144].

A promising target for future scaffold-based interventions is the liver’s neuroimmune axis, which orchestrates immune cell activation, cytokine gradients, and fibrogenesis. Scaffold-mediated modulation of the neuroimmune environment has been demonstrated in neural and peripheral nerve models, where PLGA microspheres embedded in hydrogels have enabled the controlled release of neurotrophic factors (e.g., NGF, GDNF) alongside anti-inflammatory cytokines (e.g., IL-10) to support neurite extension and immune modulation [145,146,147].

In liver models, microRNA-loaded nanoparticles (e.g., miR-29, miR-122 targeting TGF-β/SMAD pathways) have been successfully used to suppress hepatic stellate cell activation and fibrogenesis, facilitating a pro-regenerative microenvironment; however, their integration within biomaterial scaffolds to enable localised, sustained delivery in liver TE remains an open area for exploration [139,148,149]. Additionally, stimuli-responsive biomaterials that react to ROS or pH changes in inflamed hepatic environments are being developed to enable spatiotemporally controlled release of therapeutic agents, creating dynamic bioresponsive platforms for liver TE [150,151].

Although neuromodulatory agents such as capsaicin analogues and cholinergic agonists have been proposed to modulate neuroimmune pathways in MASLD and ALD models, their delivery through engineered scaffolds remains to be explored [152,153]. Advancing these strategies could enable the development of dynamic liver TE platforms for neuromodulatory interventions, within a controllable 3D environment, paving the way for next-generation regenerative and disease-modelling systems.

Third, advanced biofabrication strategies appear to be a key aspect to enable the architectural recreation of the liver’s sinusoidal networks while facilitating the integration of neural and immune components, crucial for modelling the liver’s neuroimmune-vascular complexity [154,155,156].

3D bioprinting technologies have advanced to allow for the creation of perfusable, sinusoid-like microchannel networks that establish oxygen and nutrient gradients essential for hepatocyte zonation and function [157,158]. For instance, ink printing techniques have been used to fabricate microvascular networks that support hepatocyte viability and functionality [157,158]. Co-printing endothelial cells within advanced hydrogel constructs has been shown to improve vascular barrier integrity and responsiveness to inflammatory stimuli, as demonstrated in core–shell bioprinting of vascularized in vitro liver sinusoid models and in multiscaled hepatic lobule constructs with integrated vascular networks [159,160].

These studies underscore the potential for building more physiologically relevant, neuroimmune-vascular liver TE platforms. The field of biofabrication is advancing toward the integration of neural elements within engineered constructs, particularly in peripheral nerve and spinal cord models, where nerve guidance conduits and co-culture with neural progenitors have been employed to investigate the impact of innervation on endothelial behaviour and immune modulation [161,162]. For example, biomaterials bioprinted incorporating Schwann cells and mesenchymal stem cells co-cultures have successfully guided neurite outgrowth in a 3D-printed fibrin matrix, demonstrating that bioprinting can guide axonal regeneration [163].

Although not yet applied in liver TE, these strategies hold significant translational potential for developing advanced liver models to study the neuroimmune–vascular interface and its role in hepatic regeneration and disease.

**Figure 3:**
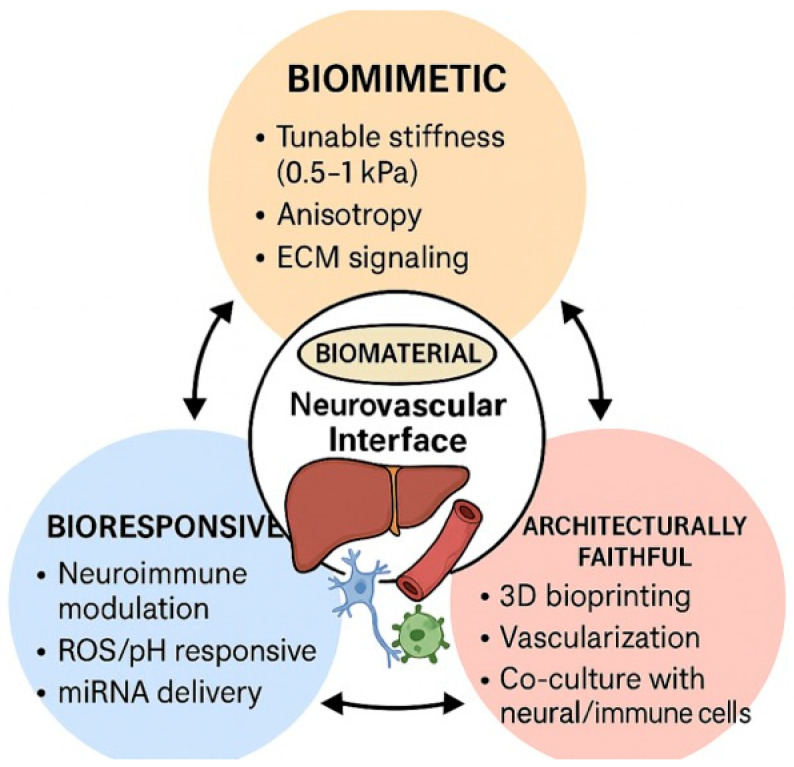
Schematic representation of the three essential design models of next-generation biomaterials for liver tissue engineering targeting the neurovascular interface.

In summary, advancing biomaterials for liver TE requires a paradigm shift from inert scaffolds toward constructs that are: (1) biomimetic, with physicochemical and mechanical properties aligned with the liver’s neuroimmune-vascular physiology; (2) bioresponsive, capable of delivering neuromodulatory and immunomodulatory factors to guide regeneration; and (3) architecturally faithful, using advanced biofabrication strategies to incorporate neural, vascular, and immune elements within engineered tissues (Figure 3). These next-generation biomaterials hold promise for developing physiologically relevant liver models to dissect neuroimmune–liver interactions and to test neuromodulatory therapies. Integrating these advanced biomaterials with AI-based digital twins and organoid systems can further enhance predictive modelling, enabling the identification of novel therapeutic targets and accelerating translational strategies in liver regenerative medicine.

## 5. Research Gaps and Future Directions

Despite growing recognition of the liver’s complex innervation and its role in neuroimmune and neurovascular regulation, the molecular mechanisms underlying the interaction between hepatic nerves and endothelial cells, particularly involving pathways such as ephrin signalling, thrombosis, remain poorly understood. While ephrins are known to mediate neurovascular alignment and axon guidance in other organs, their specific roles in liver development, regeneration, and disease progression are underexplored.

The literature on human liver innervation remains limited. Species-specific differences in hepatic innervation patterns complicate translational research, and human-focused studies using advanced imaging and organoid models are urgently needed to bridge this gap. Although insights can be extrapolated from animal models, it is well established that significant species-specific differences exist in hepatic innervation patterns. This scarcity of human-focused studies is not unexpected, given the inherent challenges in visualising nerve fibres in routine histopathological sections and the technical difficulties in designing studies capable of reliably characterising the anatomical, functional, and spatial properties of hepatic innervation in humans.

Future research should focus on human studies and translational models to bridge these gaps. Integrating neuroimaging, electrophysiology, 3D cultures, biomaterials, and omics approaches will provide a more comprehensive understanding of neural–liver interactions.

Personalised neuromodulation therapies, tailored to individual patients’ neural profiles, represent a promising avenue for future research. These therapies have the potential to revolutionise the treatment of liver diseases by providing targeted and effective interventions.

## 6. Conclusions

Liver innervation is a central yet historically underappreciated regulator of hepatic physiology and pathology. This review highlights the intricate interplay between neural, immune, and vascular systems in the liver, revealing how autonomic and sensory fibres influence inflammation, regeneration, fibrosis, and tumour progression. Emerging evidence suggests that neural signals modulate hepatic stellate cell activity, cholangiocyte proliferation, and endothelial function, underscoring the therapeutic potential of neuromodulation.

Innovative platforms such as liver organoids, AI-based digital twins, and biomimetic scaffolds offer promising avenues to model and manipulate neural–liver interactions. However, significant gaps remain—particularly in understanding neurovascular signalling and translating animal findings into human physiology. Addressing these gaps through interdisciplinary and new technological research will be key to unlocking novel therapeutic strategies for liver diseases.

## Figures and Tables

**Table 1:** Summary of hepatic innervation roles in major liver diseases. The table compares sympathetic, parasympathetic, and sensory contributions across different conditions. It highlights key mechanisms including adrenergic and cholinergic signalling, neuroimmune modulation, and neurogenic inflammation, emphasising their impact on fibrosis, regeneration, metabolic regulation, and tumour progression.

Disease	Sympathetic Role	Parasympathetic Role	Sensory/Other Notes
Liver Fibrosis	Targets sinusoids & HSCs; promotes fibrosis; α2-AR antagonists reduce HSC activation	Supports regeneration; cholinergic fibres around ductular reactions; ACh modulates HSC proliferation	CGRP from sensory fibres promotes regeneration
MASLD/MASH	Overactivity leads to metabolic dysregulation & inflammation; Endoplasmic Reticulum stress in CNS circuits leads to increased sympathetic tone	Vagal afferents regulate satiety & energy; dysfunction worsens MASLD/MASH	Gut–liver axis: serotonin (5-HT) signalling implicated
ALD	Sympathetic drive via HPA axis leads to Kupffer activation, fibrosis, NF-κB activation	Vagus modulates neuroimmune response; potential therapeutic target (VNS)	ROS & acetaldehyde sensitise the liver to immune-mediated damage
Autoimmune Liver Diseases	Norepinephrine disrupts immune tolerance; increased dendritic cell activation	Vagus promotes immune tolerance via α7nAChR; VNS reduces inflammation	Sensory fibres mediate pruritus via TGR5 & MRGPR signalling
Hepatic Malignancies	Promotes tumour growth & immune suppression via β1-AR	Anti-inflammatory, but may dampen anti-tumour immunity	Neuroimmune axis shapes tumour microenvironment

## Data Availability

No new data were created or analysed in this study. Data sharing is not applicable to this article.

## Abbreviations

The following abbreviations are used in this manuscript:

AI	Artificial Intelligence
AIH	Autoimmune Hepatitis
ALD	Alcohol-Associated Liver Disease
ALI	Acute Liver Injury
ALN	Alcohol related peripheral neuropathy
AR	Adrenergic Receptor
AUD	Alcohol Use Disorder
BBB	Blood–brain barrier
BDL	Bile Duct Ligation
BMAL1	Brain and Muscle ARNT-Like 1
CCL2	Chemokine (C-C motif) Ligand 2
CCL4	Carbon Tetrachloride
CD8	Cluster of Differentiation 8
CGRP	Calcitonin Gene-Related Peptide
CLR	Calcitonin Receptor-Like Receptor
CNS	Central Nervous System
COVID	Coronavirus Disease
DILI	Drug-Induced Liver Injury
DMV	Dorsal Motor Nucleus of the Vagus
DNA	Deoxyribonucleic Acid
DVC	Dorsal Vagal Complex
ECM	Extracellular Matrix
EPO-R	Erythropoietin Receptor
ER	Endoplasmic Reticulum
ERK1/2	Extracellular Signal-Regulated Kinases 1/2
FGF21	Fibroblast Growth Factor 21
GDNF	Glial Cell Line-Derived Neurotrophic Factor
GLP-1	Glucagon-Like Peptide 1
HCC	Hepatocellular Carcinoma
HLA-G	Human Leukocyte Antigen G
HPA	Hypothalamic–Pituitary–Adrenal Axis
HPC	Hepatic Progenitor Cell
HSC	Hepatic Stellate Cell
HT	Hydroxytryptamine (Serotonin)
HVN	Hepatic Vagus Nerve
IL	Interleukin
iPSCs	induced Pluripotent Stem Cells
MASH	Metabolic Dysfunction-Associated Steatohepatitis
MASLD	Metabolic Dysfunction-Associated Steatotic Liver Disease
MHC	Major Histocompatibility Complex
NAFLD	Non-Alcoholic Fatty Liver Disease
NASH	Non-Alcoholic Steatohepatitis
NE	Norepinephrine
NF-κB	Nuclear Factor Kappa B
NGF	Nerve Growth Factor
NTF3	Neurotrophin-3
NTRK3	Neurotrophic Tyrosine Kinase Receptor 3
PBP	Peribiliary Plexus
PCNA	Proliferating Cell Nuclear Antigen
PDTs	Patient Digital Twins
PH	Partial Hepatectomy
PLC	Phospholipase C
PLGA	Poly(lactic-co-glycolic acid)
PVN	Paraventricular Nucleus
RAMP1	Receptor Activity-Modifying Protein 1
REV-ERBα/β	Nuclear Receptors Regulating Circadian Rhythm
ROS	Reactive Oxygen Species
RYGB	Roux-en-Y Gastric Bypass
SARS-CoV-2	Severe Acute Respiratory Syndrome Coronavirus 2
SFO	Subfornical Organ
SMAD	Mothers Against Decapentaplegic Homolog
STAT5	Signal Transducer and Activator of Transcription 5
TAZ	Transcriptional Coactivator with PDZ-Binding Motif
TE	Tissue Engineering
TGF-β	Transforming Growth Factor Beta
TGR5	G Protein-Coupled Bile Acid Receptor
TH	Tyrosine Hydroxylase
VEGF	Vascular Endothelial Growth Factor
VEGF-C/D	Vascular Endothelial Growth Factor C/D
VEGFR3	Vascular Endothelial Growth Factor Receptor 3
VIP	Vasoactive Intestinal Peptide
VLDL	Very-Low-Density Lipoprotein
VNS	Vagus Nerve Stimulation
YAP	Yes-Associated Protein
